# Soil microclimate and vegetation dynamics shape elevational and seasonal variations of diazotrophic communities in alpine grasslands

**DOI:** 10.3389/fpls.2025.1587343

**Published:** 2025-09-15

**Authors:** Junpeng Rui, Xiaojian Long, Xuemiao Wang, Xinyu Xiong, Jianxiao Zhu

**Affiliations:** ^1^ State Key Laboratory of Herbage Improvement and Grassland Agro-ecosystems, College of Ecology, Lanzhou University, Lanzhou, China; ^2^ Key Laboratory of Biodiversity and Environment on the Qinghai-Tibetan Plateau, Ministry of Education, School of Ecology and Environment, Tibet University, Lhasa, China; ^3^ Center for Grassland Microbiome, Lanzhou University, Lanzhou, China; ^4^ Gannan Tibetan Autonomous Prefecture Academy of Agriculture, Forestry and Animal Husbandry Grassland Science, Gannan, China; ^5^ College of Pastoral Agriculture Science and Technology, Lanzhou University, Lanzhou, China

**Keywords:** diazotroph, alpine grassland, altitudinal gradient, seasonal dynamics, nitrogen fixation, *nifH* gene, climate change, plant biomass

## Abstract

**Introduction:**

Diazotrophs play critical roles in maintaining ecosystem nitrogen (N) cycling in alpine grasslands. However, the elevational and seasonal variations of diazotrophic communities in these ecosystems remain poorly understood. This gap in knowledge limits our ability to predict how N fixation will respond to environmental change. Here, we investigated the seasonal dynamics of soil diazotrophic communities across a 3200-4000 m elevational gradient in Qinghai-Tibetan alpine grasslands during the growing season.

**Methods:**

Soil samples were collected across an elevational gradient (3200-4000 m) throughout the growing season. The diazotrophic community composition was assessed by sequencing the nifH gene, which was also quantified using quantitative PCR. Soil nitrogenase activity was measured to assess N fixation potential. Key environmental variables, such as soil temperature, moisture, and plant biomass (particularly legume biomass), were monitored.

**Results and Discussion:**

Our results revealed that diazotrophic alpha-diversity followed an inverted V-shaped pattern along the elevational gradient, primarily driven by soil temperature and moisture. Beta-diversity analyses demonstrated that diazotrophic communities generally exhibited similar elevational distribution patterns throughout the growing season, also primarily influenced by temperature and moisture. Seasonal variations in diazotrophic communities were more pronounced at lower elevations, primarily associated with plant biomass dynamics, including delayed legume emergence at 3200 m in June and their subsequent biomass accumulation after July. In contrast, soil microclimate (particularly temperature) dominated community shifts at higher elevations. Notably, *nifH* gene abundance and soil nitrogenase activity were higher in the early growing season, suggesting free-living diazotrophs may play a crucial role in N fixation. Abundant species were key contributors to diazotrophic beta-diversity. Symbiotic *Mesorhizobium* was more abundant at low elevations, while free-living *Geobacter* at high elevations. Conversely, associative diazotrophs peaked later in the growing season, in contrast to *Geobacter*. Rare species played a key role in shaping alpha diversity, particularly at mid-elevations, where soil moisture was the highest. Our study underscores the complex interactions between soil microclimate change and plant dynamics in regulating diazotrophic communities. Furthermore, it highlights the essential roles of both abundant and rare species in sustaining ecosystem functions in alpine grasslands. These findings provide new insights into the biogeochemical processes supporting N cycling in alpine grasslands and highlight the potential impacts of vegetation and climate change on these fragile ecosystems.

## 1 Introduction

Biological nitrogen fixation (BNF) is a crucial process in terrestrial ecosystems that transforms atmospheric N_2_ into plant-available nitrogen (N) (Herridge et al., 2008; Vitousek et al., 2013). N-fixing microorganisms comprise three types: symbiotic, associative, and free-living diazotrophs. Symbiotic and associative diazotrophs exhibit superior N fixation efficiency and have been the subject of more comprehensive research. Free-living diazotrophs, though less efficient, are extensively spread across ecosystems and are essential for N intake in N-limited environments (Houlton et al., 2008). Understanding the distribution and function of these N-fixing types across different ecosystems is critical for revealing the mechanisms of N cycling, especially in extreme environments such as alpine grasslands (Yang et al., 2014).

The diversity and N fixation activity of soil diazotrophic communities are regulated by multiple environmental factors, which can be categorized into climatic, edaphic, and biotic drivers. Among these, temperature and water conditions (e.g., precipitation, soil moisture) are recognized as the two most critical determinants, particularly in shaping large-scale distribution patterns (Zhao et al., 2020). In cold environments, low temperatures constrain nitrogenase activity, while warming can enhance enzymatic efficiency and subsequently reshape diazotrophic community structure (Rousk et al., 2017). Soil moisture influences diazotrophs both directly (by modulating microbial growth and metabolism) and indirectly (by altering soil oxygen diffusion, given the well-documented oxygen sensitivity of nitrogenase) (Smercina et al., 2021). Elevated soil available N (e.g., ammonium, and nitrate) suppresses N fixation, with free-living diazotrophs being particularly sensitive due to their preferential uptake of environmental N sources (Dynarski and Houlton, 2018). In contrast, symbiotic N fixation relies on legume-rhizobia interactions within root nodules (Holzmann and Haselwandter, 1988), while associative fixation depends on plant hosts (e.g., grasses) (Wickstrom and Garono, 2007). Consequently, shifts in plant community composition (including species identity and biomass) may significantly alter the structure and function of soil diazotrophic communities. Despite this mechanistic understanding of individual drivers, how these factors collectively regulate diazotroph communities remains a critical gap in alpine ecosystem ecology.

Alpine grasslands, situated in high-elevation regions, provide essential ecological services, including water regulation, and biodiversity preservation (Körner, 2004). These ecosystems are exceptionally sensitive to climate change, characterized by low temperatures, rendering biodiversity and ecological processes more vulnerable (Shangguan et al., 2024). The elevational gradient serves as an important platform for studying climate change impacts, differing elevations lead to substantial alterations in environmental variables including temperature and water conditions (e.g., precipitation and soil moisture) (Lomolino, 2001; Sundqvist et al., 2013). These changes not only affect plant growth but also have profound effects on microbial community structure and function. For instance, low-elevation areas are typically warmer, hence facilitating the proliferation of leguminous plants and potentially enhancing symbiotic N fixation (Tang et al., 2024).

Seasonal changes are crucial determinants of microbial community dynamics in alpine grasslands. High-elevation ecosystems typically exhibit a short growing season and experience large temperature fluctuations, resulting in a dramatic effect of seasonal shifts on microbial communities (Zhao and Hu, 2023). Changes in factors such as plant growth, temperature, and precipitation throughout a growing season may influence the structure and function of diazotrophic communities. For example, in early growing season, characterized by low temperatures and sluggish growth of leguminous plants, symbiotic N fixation may exhibit reduced efficiency (Chellem et al., 2024). Plants have diverse N requirements at different stages of their growth cycle (Wang et al., 2024). Furthermore, the N-fixing capacity of legume root nodules also fluctuates (Daubech et al., 2017). Consequently, the seasonal fluctuations of various diazotroph types necessitate additional examination.

Microbial communities exhibit a characteristic structure where abundant species dominate in terms of biomass and functional contributions, while rare species, despite their low abundance, can play critical roles in maintaining community resilience and adaptability (Litchman et al., 2024). Despite these general trends, the specific roles of abundant and rare species in shaping microbial community diversity remain an area of active debate in the field (Ma et al., 2024; Ye et al., 2024). Abundant species in diazotrophic community, such as rhizobia and associative diazotrophs, have received more attention. However, despite the remarkably high diversity of soil diazotrophs, the ecological roles of rare species (e.g., numerous soil free-living diazotrophs) remain poorly understood. How they contribute specifically to diazotrophic communities in alpine ecosystems, in terms of both alpha and beta diversity, is still poorly understood and warrants further investigation.

Understanding the elevational-seasonal dynamics of diazotrophic communities is critical for predicting alpine ecosystem responses to climate change, as it may determine the stability of nitrogen inputs in these vulnerable systems. However, two key knowledge gaps remain: (1) whether the elevational patterns of diazotrophs persist throughout the growing season, given that most existing data come from single-timepoint surveys (Rui et al., 2022); and (2) how the relative importance of soil microclimatic (e.g., soil temperature and moisture) versus biotic (e.g., plant biomass) controls shifts across elevations.

Here, we address these gaps by systematically investigating soil diazotrophic communities along a 3200-4000 m elevational gradient during the growing season (June-September) in Qinghai-Tibetan alpine grasslands. We aim to answer three scientific questions: (1) Do diazotrophic communities exhibit consistent elevational distribution patterns throughout the growing season, and what are the key factors? (2) Are seasonal dynamics of diazotrophic communities elevation-dependent, and how do the controlling factors (e.g., soil microclimate vs. vegetation dynamics) vary with elevation? (3) What are the distinct roles of abundant versus rare species in maintaining diazotrophic community diversity across spatial and temporal scales? Our findings will provide mechanistic insights into how climate-vegetation interactions regulate N fixation potential in alpine ecosystems under rapid climate change.

## 2 Materials and methods

### 2.1 Study site description and soil sampling

The study site is located at the Haibei National Field Research Station of Alpine Grassland Ecosystem (101°12’E, 37°37’N, approximately 3200 m *a.s.l.*) on the northeastern Qinghai-Tibetan Plateau in Qinghai, China. The region experiences an annual average air temperature of -1.1°C and an average annual precipitation of 485 mm. More than 80% of the annual rainfall occurs in the growing season (Yang et al., 2014), which lasts from May to September. Due to persistent snow cover in high-elevation areas in early May, we collected soil samples at the beginning of June, July, August, and September 2021. The monthly precipitation at 3200 m during these months were 90, 64, 125, and 104 mm, respectively (Rui et al., 2023). Unfortunately, the precipitation data for the higher elevation sites were not collected.

The experimental plots were established at five elevations (3200, 3400, 3600, 3800, and 4000 m *a.s.l*). At each elevation, six 1 m × 1 m quadrats were designated as replicates with an interval of 10 m. The soils, classified as Mat-Gryic Cambisol, are primarily vegetated with perennial flora. Geographic coordinates and vegetation features for these elevations are available in our previous study (Rui et al., 2022). Five soil cores (0-10 cm depth) were randomly obtained from each quadrat, amalgamated into a single sample, and sieved through a 2-mm mesh. Concurrently, aboveground plant biomass and species richness were documented, with particular attention to leguminous species which were separately collected and weighed to quantify their biomass contribution. Soil temperature was measured with EM-50 devices (Decagon devices, USA) at a depth of 5 cm, and the average monthly temperature was computed. Methods for measuring soil moisture, pH, ammonium, and nitrate contents were reported previously (Rui et al., 2022). Soil C:N ratio denotes the ratio of total carbon to total N. Soil nitrogenase activity was determined using the acetylene reduction assay (Hardy et al., 1973).

### 2.2 DNA extraction, qPCR and sequencing

Genomic DNA was extracted from 1 g fresh soils using the PowerSoil DNA Isolation kit (MO BIO Laboratories, USA) in accordance with the manufacturer’s guidelines. The universal *nifH* primers PolF (5’-TGC GAY CCS AAR GCB GAC TC-3’) and PolR (5’-ATSGCCATCATYTCRCCGGA-3’) were employed for amplicon sequencing and quantitative PCR (qPCR), adhering to the procedure established by Che et al (Che et al., 2018). Sequencing libraries were constructed using TruSeq^®^ DNA Kit (Illumina, USA), and sequencing was conducted on the Illumina NovaSeq platform (Novogene, Beijing, China) with the Reagent Kit v2 (2 × 250 bp).

### 2.3 Sequencing data analysis

Paired-end reads were merged using FLASh v1.2.11 (Magoč and Salzberg, 2011). Primers were eliminated from the merged reads using the Perl script *trim_primer_in fq.pl*. Low quality reads and chimeras were eliminated utilizing Trimomatic v0.39 (Bolger et al., 2014) and Uchime, respectively. Frameshifts were corrected using FrameBot (Wang et al., 2013), and sequences that cannot be corrected through frameshift correction were removed. Amplicon Sequence Variants (ASVs) were deduced from the nucleotide sequences employing the Unoise algorithm (Edgar, 2016). Representative ASV sequences were aligned against the local BLAST database mentioned before (Rui et al., 2022), and their taxonomic affiliations were ascertained using lowest common ancestor (LCA) algorithm in MEGAN 6 (Huson et al., 2016). Based on existing literature, diazotrophic genera were classified into three groups: symbiotic, associative, and free-living diazotrophs (**Supplementary Table S1**). For the ASV table, sequences from each sample were randomly subsampled to the same sequence depth (16227 reads per sample in this study) using the Perl script *subsample_in_table.pl.* ASVs were categorized as abundant (>0.1% average relative abundance across all samples), common (0.01–0.1%), or rare (<0.01%). Alpha-diversity indices, including observed species and Shannon diversity, were calculated with the script *alpha_diversity.pl*. All of the Perl scripts mentioned above have been described previously (Rui et al., 2023).

### 2.4 Statistical analyses

Generally, statistical analyses based on the ASV table and environmental factors were predominantly conducted using R (version 4.3.3) as outlined by Rui et al (Rui et al., 2022). To evaluate differences in diazotrophic communities across elevations or months, ANOVA with Tukey’s *post hoc* test was applied using the *aov* function from *vegan* package and *HSD.test* from *agricolae*. Non-metric multidimensional scaling (NMDS) based on Bray-Curtis dissimilarity was performed using *metaMDS* from *vegan*, with environmental variables incorporated into the NMDS plots by the *envfit* function.

Community differences across elevational and seasonal gradients were evaluated using the PerMANOVA (i.e., Adonis) test through the *adonis2* function. Based on Bray-Curtis dissimilarity matrices, two-way Adonis design was implemented to assess the individual and interactive effects of elevation and season on diazotrophic community composition. One-way Adonis was used to assess the pairwise differences between specific elevations and monthly time points. The analysis employed 999 permutations to ensure robust significance testing.

Partial Mantel test was conducted using the *ecodist* package to assess the influence of environmental factors on community variances. When evaluating each factor’s influence, the remaining factors were treated as covariates to isolate the independent effect.

We analyzed the effects of environmental factors on N-fixation related variables (alpha diversity, nitrogenase activity, *nifH* gene abundance, and relative abundances of specific diazotrophs) using linear mixed-effects models implemented in the *lme4* package, with plot as random effect. Temperature, soil moisture, plant biomass and soil properties were included as fixed effects. To obtain standardized effect sizes (β coefficients), all continuous predictors were z-score normalized (mean-centered and scaled by 1 standard deviation) prior to model fitting. Separate models were run to examine either elevational gradients (controlling for seasonal variation) or seasonal dynamics (controlling for elevation), respectively. The relative importance of each predictor was quantified using variance partitioning analysis through the *glmm.hp* package (Lai et al., 2022). This approach allowed us to assess both the direction and magnitude of environmental effects while accounting for spatial autocorrelation among plots.

## 3 Results

### 3.1 Plant dynamics and *nifH* gene abundance/nitrogenase activity

Aboveground biomass showed clear seasonal patterns across elevations (**Supplementary Figure S1H**), with consistently lower values in June (initial growth phase) than in July–September. Legumes (primarily *Oxytropis*, *Tibetia himalaica* and *Medicago*), representing <2.3% of total biomass, were scarce at higher elevations but increased substantially at 3200 m from July onward, after minimal presence in June (**Supplementary Figure S1I**).

The abundance of *nifH* genes (i.e., number of *nifH* gene copies) for each month exhibited an inverted V-shaped pattern along the elevational gradient, peaking at 3600 m (**Supplementary Figure S1J**), with significantly higher values in June compared to later months (July-September), particularly at 3600 m and 3200 m. There was no significant difference in *nifH* gene abundance between July and September. Across all samples, the abundance of *nifH* genes showed a significant positive correlation with soil moisture (Spearman’s ρ = 0.585, *P*<0.001), and a significant negative correlation with legume biomass (Spearman’s ρ = -0.677, *P*<0.001).

**Figure 1:**
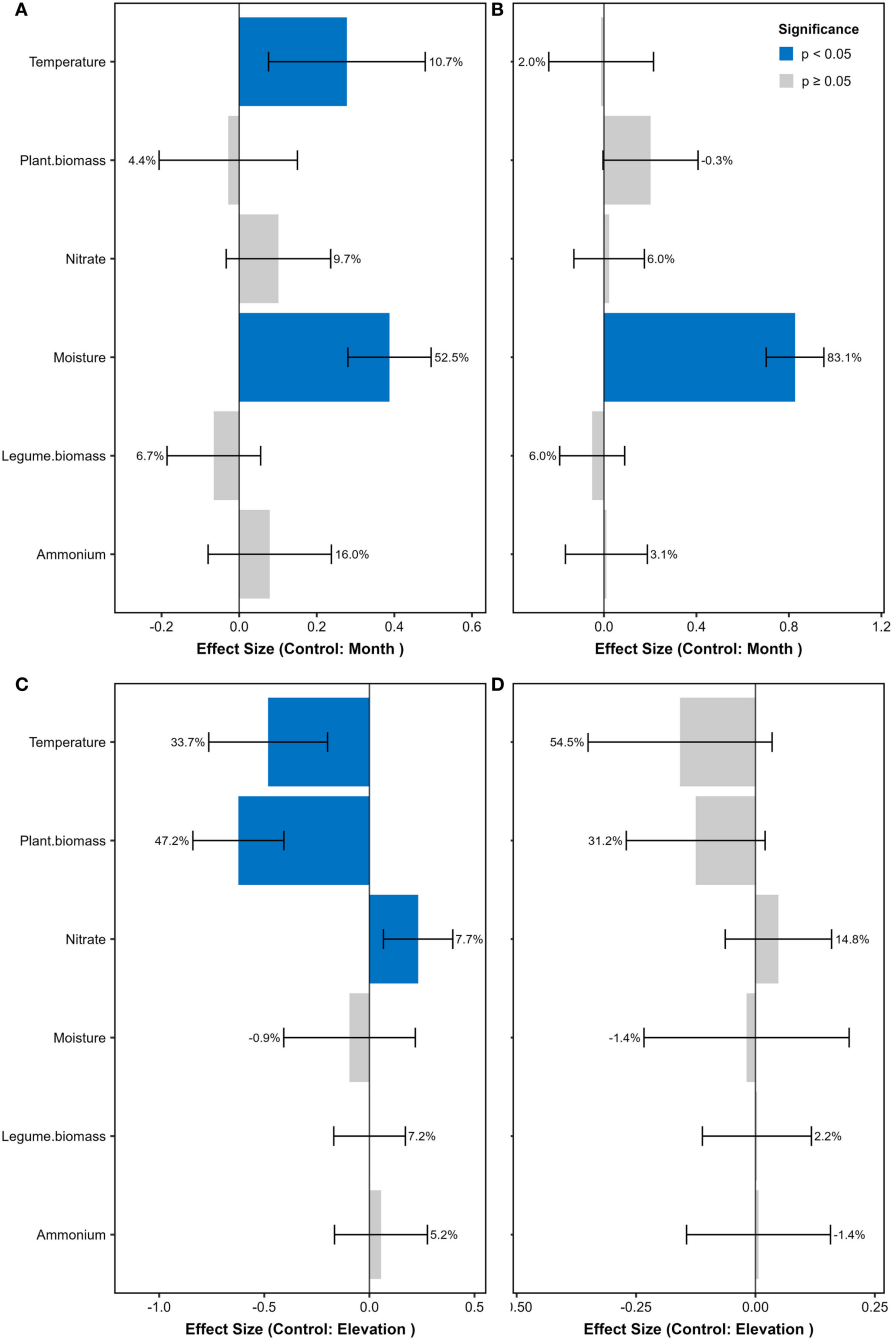
Standardized effects on nitrogenase activity and *nifH* gene abundance from linear mixed-effects models. **(A)** Nitrogenase activity patterns along elevational gradients (controlling for seasonal variation by month). **(B)** *nifH* gene abundance patterns along elevational gradients (controlling for season). **(C)** Nitrogenase activity seasonal dynamics (controlling for elevation). **(D)** *nifH* gene abundance seasonal dynamics (controlling for elevation). Predictors were z-score standardized prior to analysis. Blue bars indicate significant effects (*P* < 0.05; error bars show 95% CI), with adjacent percentages representing the proportion of variance explained by each predictor (from *glmm.hp* partitioning). Gray bars denote non-significant effects (*P* ≥ 0.05). Positive β values denote enhancing effects, negative values indicate inhibitory effects. Models were fitted using *lme4* with plot as random intercept.

The soil nitrogenase activity from July to September also displayed an inverted V-shaped pattern along the elevation gradient, reaching its peak at 3600 m (**Supplementary Figure S1K**). However, in June, it was highest at both 3200 m and 3600 m, followed by 3400 m. At each elevation, the nitrogenase activity in June was consistently higher than that from July to September. It was the lowest in the high-elevation soils in September. The abundance of *nifH* gene showed the strongest correlation with soil nitrogenase activity (Spearman’s ρ = 0.52, *P*<0.001). The linear mixed-effects model results showed that soil moisture had significant positive effects on both nitrogenase activity and *nifH* gene abundance, explaining most of their elevational variations (**Figure 1**). Temperature also showed positive effects on the elevational patterns of nitrogenase activity. In contrast, Both temperature and plant biomass exhibited significant negative effects on the seasonal variations of nitrogenase activity.

### 3.2 The soil diazotrophic community composition in alpine grasslands

In this study, a total of 5131 *nifH* gene ASVs from 11 phyla were identified, dominated by Pseudomonadota and Thermodesulfobacteriota (**Supplementary Figure S2**). ASV numbers belonging to abundant, common, and rare ASVs were 192, 1078 and 4562, respectively. Abundant, common, and rare ASVs accounted for 58.3%, 33.5% and 8.3% of total abundance, respectively. The abundant ASVs were exclusively classified into Pseudomonadota, Thermodesulfobacteriota, Myxococcota and Actinomycetota.

Pseudomonadota was the most abundant phylum, with an average relative abundance of 75%, comprising 3104 ASVs, primarily distributed in Alphaproteobacteria and Betaproteobacteria. The average relative abundance of Thermodesulfobacteriota was 21%, comprising 1286 ASVs, predominantly anaerobic free-living genus *Geobacter*. Each of the remaining phyla comprised fewer than 200 ASVs. In Myxococcota, all ASVs were exclusively assigned to the anaerobic free-living genus *Anaeromyxobacter*. Within Verrucomicrobiota, the majority of ASVs were classified under the family Opitutaceae. For Actinomycetota, most ASVs (81%) were affiliated with the genus *Frankia*, while Cyanobacteriota ASVs were predominantly members of the order Nostocales.

Symbiotic diazotrophs were dominated by *Mesorhizobium*, followed by *Bradyrhizobium*, with lower relative abundances of *Frankia*, *Rhizobium*, and *Paraburkholderia* (**Supplementary Figure S3**). Associative diazotrophs were mainly represented by *Azospirillum*, followed by *Azoarcus*. Free-living diazotrophs were dominated by *Geobacter*, with other genera with average relative abundances greater than 1% including *Hydrogenophaga*, *Anaeromyxobacter*, *Dechloromonas*, and *Azonexus* (**Supplementary Table S1**).

### 3.3 Changes in the relative abundance of diazotrophic taxa

The relative abundance of dominant diazotrophs showed consistent elevational trends throughout the growing season. Pseudomonadota, (e.g., *Azospirillum*) followed a V-shaped pattern, reaching its lowest at 3600 m. In contrast, symbiotic diazotrophs (e.g., *Mesorhizobium* and *Bradyrhizobium*) exhibited the highest relative abundance at 3200 m, with no significant variation between 3400 and 4000 m. Conversely, Thermodesulfobacteriota showed an inverted V-shaped pattern, peaking at 3600 m, with *Geobacter* as a representative genus.

Many diazotrophs showed significant seasonal dynamics in relative abundance during the growing season. Diazotrophs affiliated to Pseudomonadota, Verrucomicrobiota, and Actinomycetota were significantly lowest in June, whereas Thermodesulfobacteriota showed the opposite trend (**Supplementary Figure S2**). Myxococcota (mainly *Anaeromyxobacter*) was the highest in August, especially at 3600 m. At 3200-3400 m, Actinomycetota (mainly Frankia) was the highest in July.

Along the altitudinal gradient, symbiotic diazotrophs exhibited the highest relative abundance in soils at 3200 m (**Supplementary Figure S3**). Associative diazotrophs showed a V-shaped pattern along elevation, whereas free-living diazotrophs displayed an inverted V-shaped pattern. Compared to other months, soils in June contained higher free-living and symbiotic diazotrophs, but lower associative diazotrophs. *Azoarcus*, the second highest associative diazotroph, was very low in June (**Supplementary Figure S3E**).

**Figure 2:**
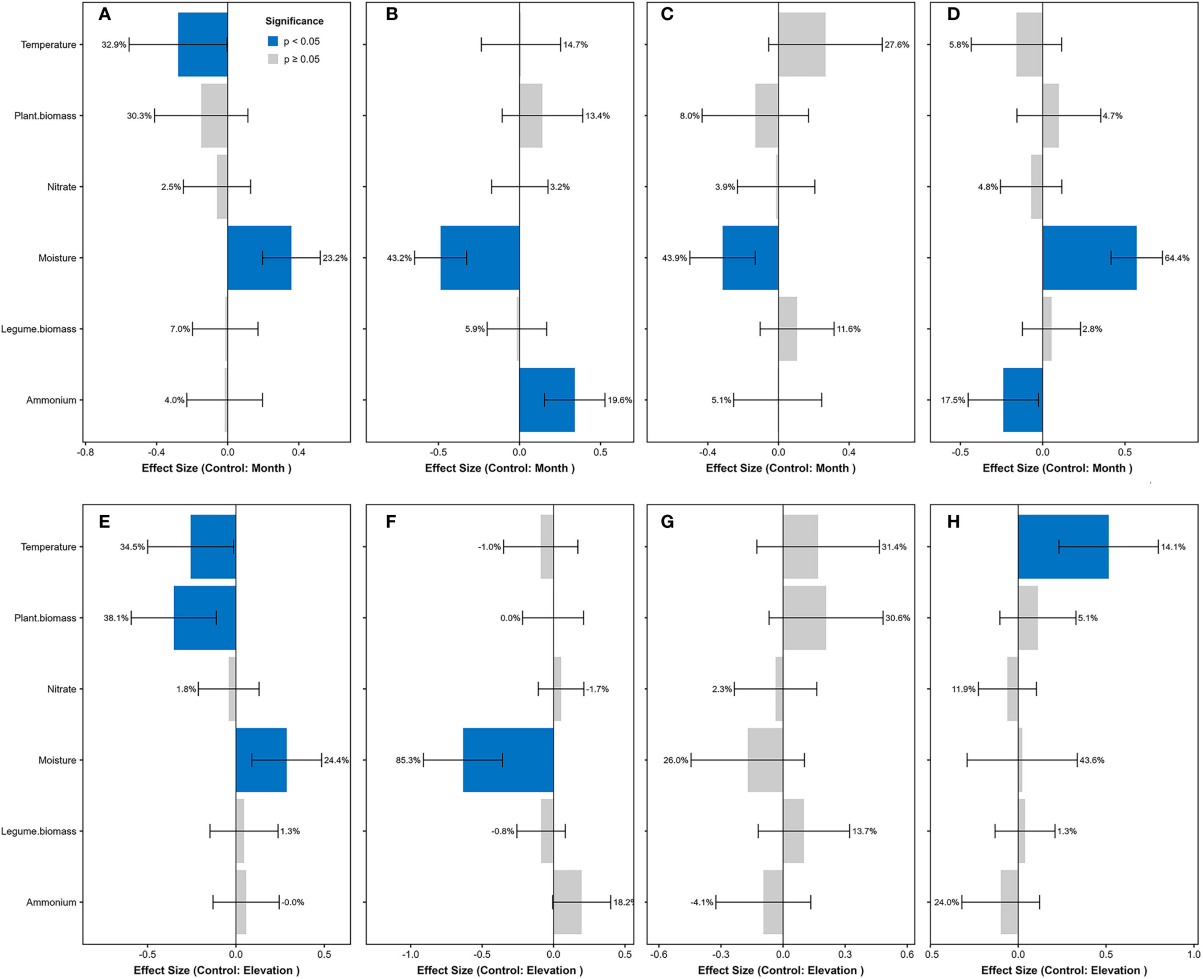
Standardized effects on diazotrophic functional groups and richness. Models controlling for seasonal variation (by month): **(A)** free-living, **(B)** symbiotic, **(C)** associative diazotrophs, and **(D)** diazotrophic richness. Models controlling for elevation: **(E)** free-living, **(F)** symbiotic, **(G)** associative diazotrophs, and **(H)** diazotrophic richness. Predictors were z-score standardized prior to analysis. Blue bars indicate significant effects (*P* < 0.05; error bars show 95% CI), with adjacent percentages representing the proportion of variance explained by each predictor (from *glmm.hp* partitioning). Gray bars denote non-significant effects (*P* ≥ 0.05). Positive β values denote enhancing effects, negative values indicate inhibitory effects. Models were fitted using *lme4* with plot as random intercept.

The linear mixed-effects models revealed that across both elevational gradients and seasonal dynamics, soil moisture showed significantly positive effects on free-living diazotrophs but negative effects on symbiotic diazotrophs (**Figure 2**). Conversely, soil temperature exhibited significantly negative effects on free-living diazotrophs and marginally positive effects on associative diazotrophs (*P* < 0.1). These results demonstrate that temperature and moisture collectively drive functional shifts in diazotrophic community structure.

### 3.4 Alpha diversity of diazotrophic community

**Figure 3:**
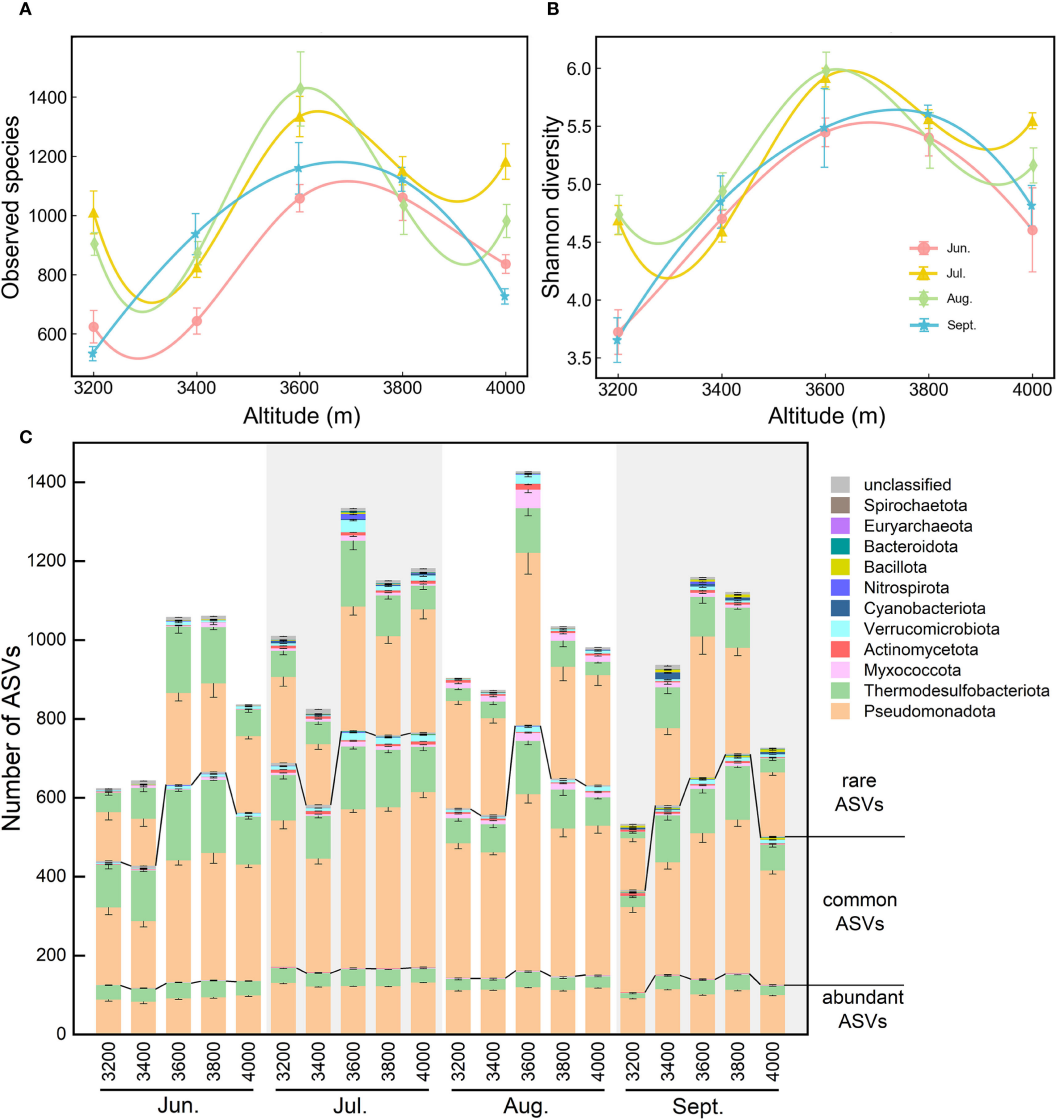
Elevational changes of alpha diversity indices during the growing season. **(A)** Observed species. **(B)** Shannon diversity. **(C)** Number of ASVs: ASVs from each elevation were sequentially classified by abundance category (abundant/common/rare ASVs), then by phylum affiliation. Error bars represent standard errors (n = 6).

Across the elevational gradient, both richness (i.e., observed species) and Shannon diversity of soil diazotrophs during the growing season followed an inverted V-shaped pattern, peaking between 3600 and 3800 m (**Figure 3**). Additionally, alpha diversity showed significant seasonal variations, higher in July and August than that in June and September, especially at 3200 and 4000 m.

Elevational and seasonal variations in alpha diversity (particularly richness) were predominantly driven by Pseudomonadota and Thermodesulfobacteriota (**Figure 3C**). Compared to abundant ASVs, common and rare ASVs contributed more to the elevational and seasonal variations in richness. For example, at low elevations (3200-3400 m) in June and September, the number of common and rare ASVs from these two phyla (especially for Thermodesulfobacteriota) was significantly lower than that at 3600-3800 m. At 3200 m in September, richness was lowest, with the lowest ASV numbers for Thermodesulfobacteriota. At 3600 m, rare ASVs of Thermodesulfobacteriota were mostly from *Geobacter*, while rare ASVs of Pseudomonadota were largely unclassified Alphaproteobacteria.

Temperature and soil moisture were the primary factors influencing the elevational pattern of diazotrophic richness, and temperature was also the primary factor affecting seasonal dynamics at most elevations (**Supplementary Figure S4**). In detail, temperature played a dominant role in June and July, whereas soil moisture was most important in August and September. At the same elevation, the main factors driving the seasonal dynamics of diazotrophic richness varied: temperature was the primary factor at 3200 m, 3600 m, and 4000 m, while aboveground biomass at 3400 m. No significant influencing factor was identified at 3800 m.

The linear mixed-effects models revealed distinct environmental controls on diazotrophic diversity. Soil moisture dominated elevational patterns (explaining 64.4% of richness variation), showing strong positive effects, while soil ammonium concentration exerted negative influences (17.5%) (**Figure 2**). Seasonally, temperature drove diversity dynamics (14.1% explained variation). These results demonstrate consistent positive effects of moisture (more spatially) and temperature (more temporally) on richness, contrasted by ammonium’s suppressive role.

### 3.5 Beta diversity of diazotrophic community

**Figure 4:**
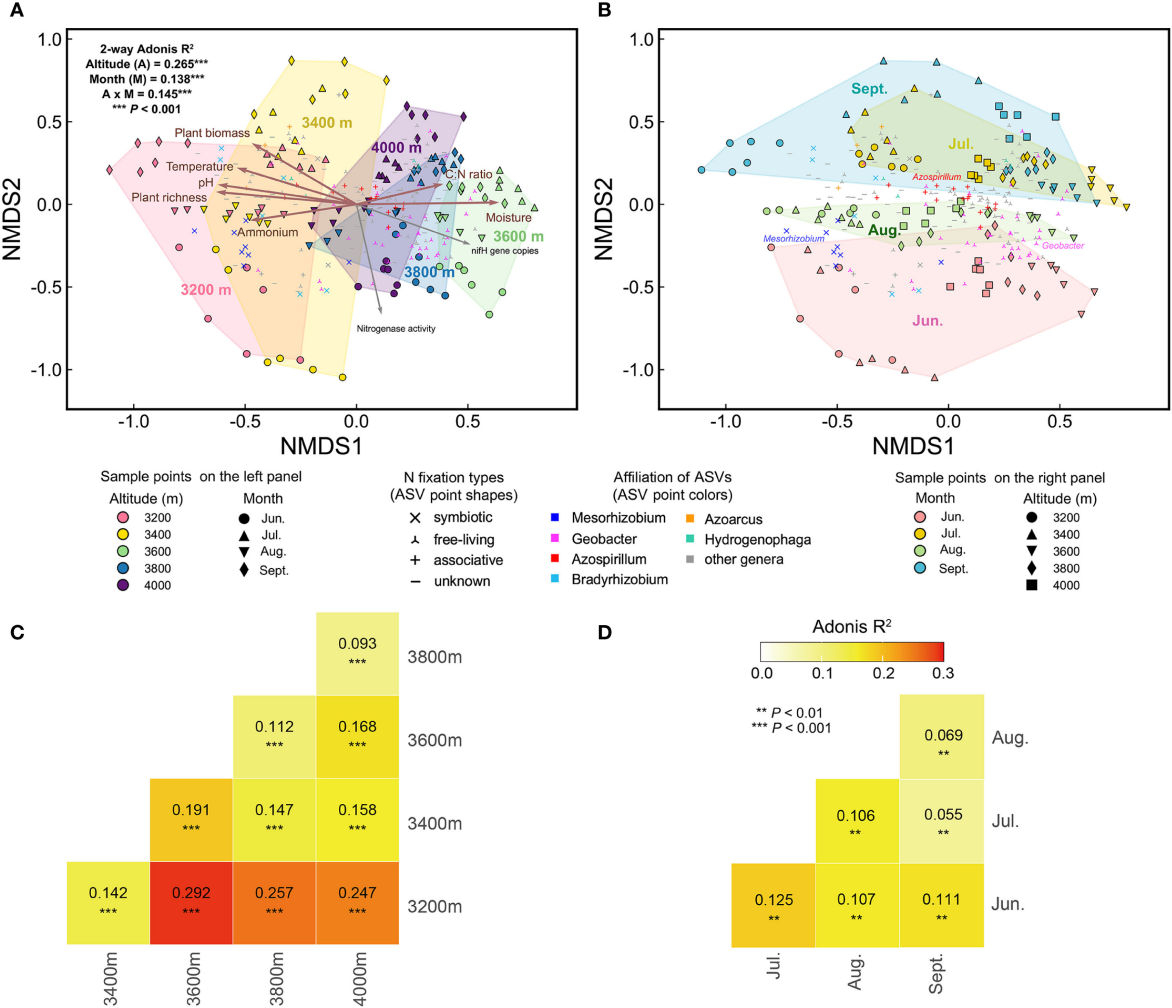
NMDS patterns and Adonis test for diazotrophic communities. **(A)** The NMDS plot colored by elevation. **(B)** The same NMDS plot colored by sampling month. **(C)** Adonis test for community differences across elevations. **(D)** Adonis test for community differences across the growing season. Environmental factors were fitted onto the NMDS plot. Brown arrows indicate factors which significantly influenced diazotrophic communities, as identified by partial Mantel test (**Table 1**). Gray arrows represent *nifH* gene copies and nitrogenase activity. Points representing abundant ASVs are displayed on the NMDS plots. Significance of Adonis test: * *P* < 0.01, ** *P* < 0.001.

**Figure 5:**
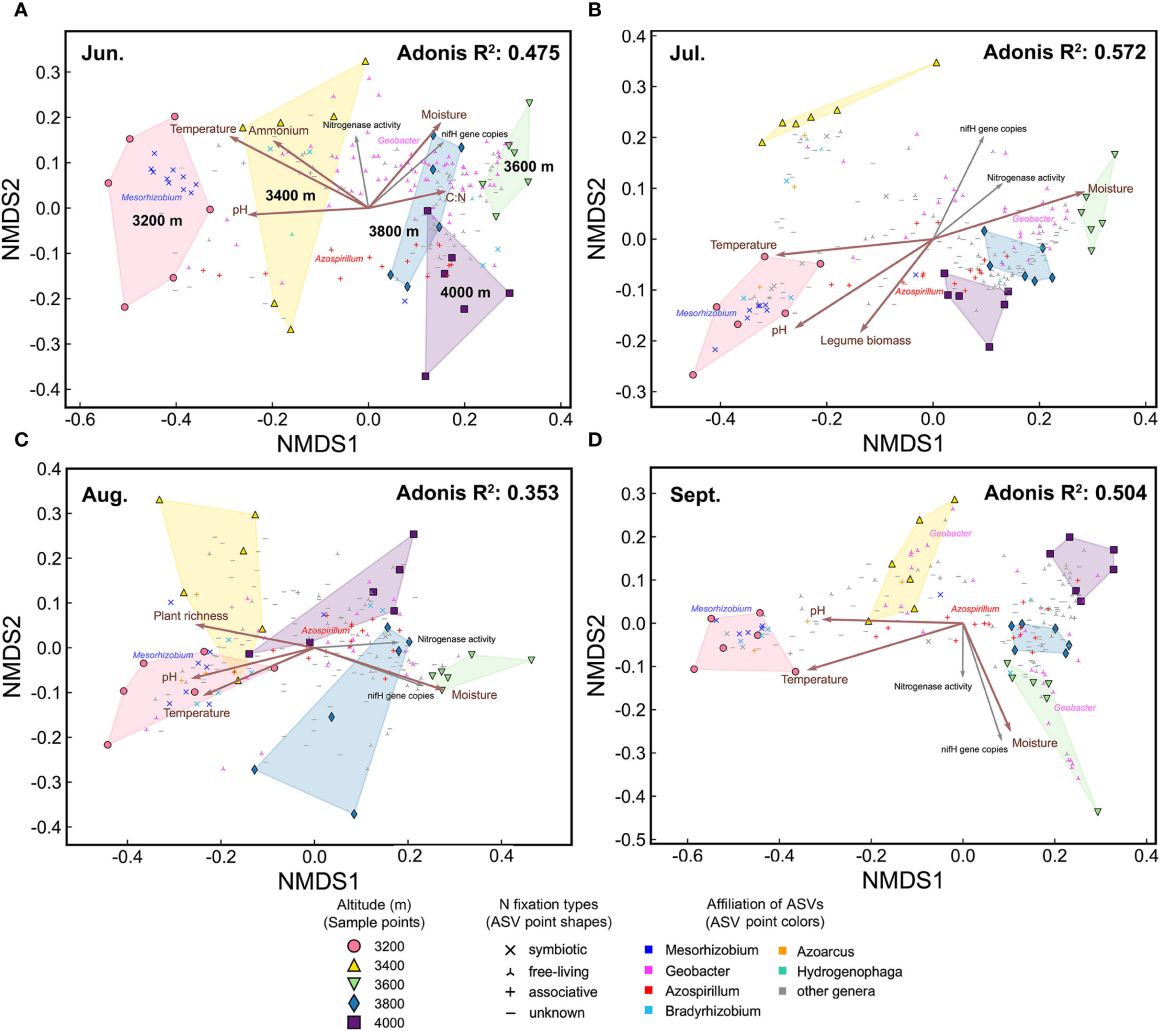
The NMDS plots of diazotrophic communities in each month. **(A)** June, **(B)** July, **(C)** August, and **(D)** September. Environmental factors are fitted onto the patterns. Brown arrows indicate factors which significantly influenced diazotrophic communities, as identified by partial Mantel test. Gray arrows represent *nifH* gene copies and nitrogenase activity. Points representing abundant ASVs are displayed on the NMDS plots. Adonis *P*<0.001.

**Figure 6:**
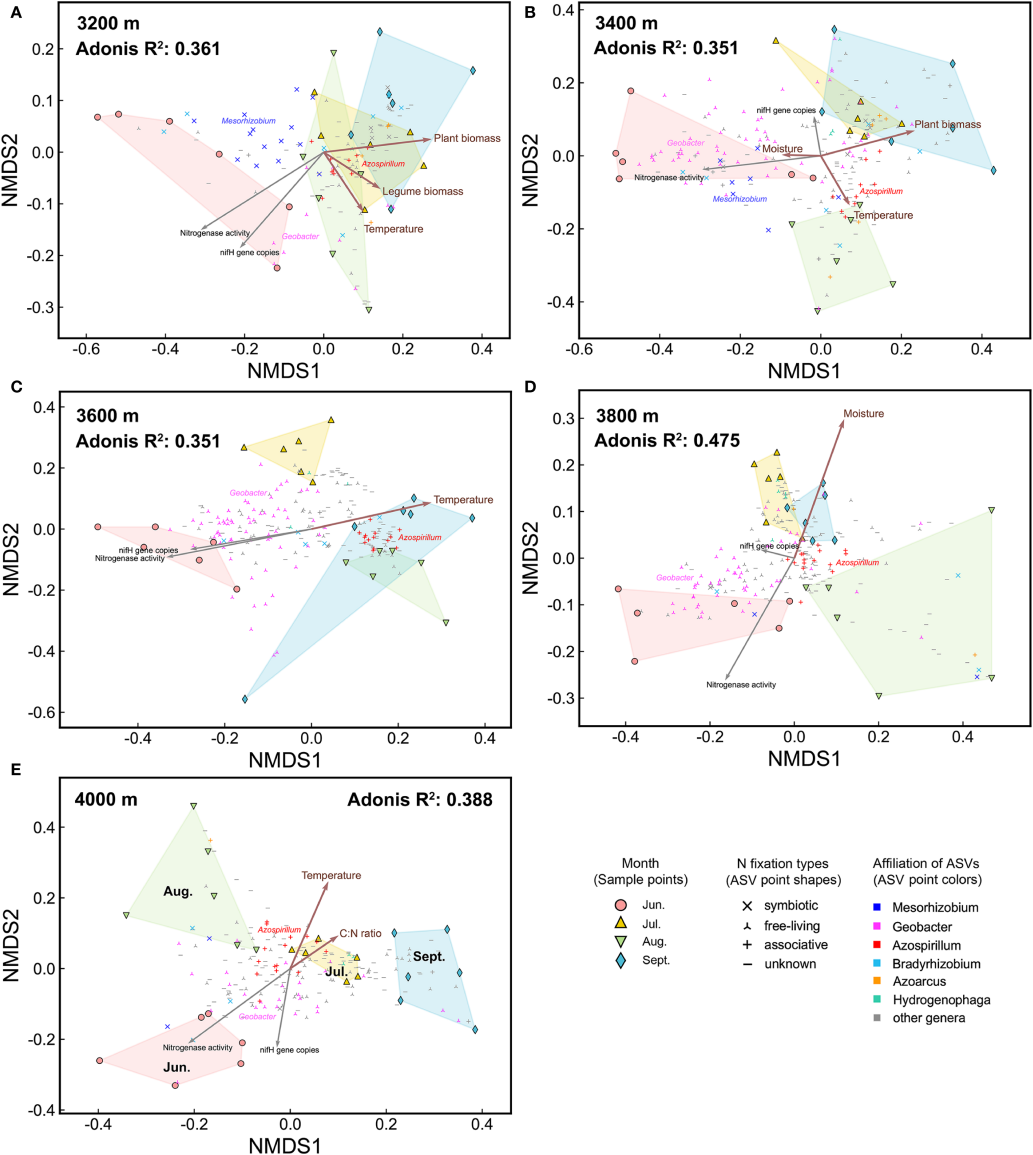
The NMDS plots of diazotrophic communities at each elevation. **(A)** 3200 m, **(B)** 3400 m, **(C)** 3600 m, **(D)** 3800 m, and **(E)** 4000 m. Environmental factors are fitted onto the patterns. Brown arrows indicate factors which significantly influenced diazotrophic communities, as identified by partial Mantel test. Gray arrows represent *nifH* gene copies and nitrogenase activity. Points representing abundant ASVs are displayed on the NMDS plots. Adonis *P*<0.001.

The beta diversity of the diazotrophic community varied significantly with elevation and season (**Figure 4**). Specifically, elevation, season (i.e., months), and their interaction explained 26.5%, 13.8%, and 14.5% of the community variation, respectively. In each month, there were significant community differences among different elevations (**Figures 5**, **4C**). Compared to 3200 m, community similarity did not decrease linearly with elevation; in other words, it did not fully conform to the distance-decay relationship. For instance, communities at 3200 m were more similar to those at 4000 m than to those at 3600 m (**Figure 4C**). At each elevation, there were also significant community differences among different months (**Figures 6**, **4D**). Seasonal dynamics of diazotrophic communities were more pronounced at low elevations (3200-3400 m), especially between June and other months (**Figure 4A**).

The distribution of abundant ASVs in NMDS plots showed that the dominant genera, such as *Mesorhizobium* and *Geobacter*, were the primary contributors to elevational and seasonal variation in beta diversity (**Figure 4**). Along the elevational gradient, ASVs belonging to *Mesorhizobium* were more abundant at 3200 m, while those belonging to *Geobacter* were more abundant at high elevations (**Figures 5**, **4A**). Seasonally, ASVs belonging to these genera were more prevalent in June, contributing to the distinct community differences between June and other months (**Figures 6**, **4B**). Additionally, ASVs of *Azospirillum* were clustered near the NMDS origin, indicating their relatively minor contribution to community differences. The V-shaped elevation changes of *Azospirillum* were most pronounced in June and July, while in August and September, the relative abundance of this genus increases at intermediate elevations, narrowing the differences between elevations. This results in the ASVs of this genus being closer to the origin position on the NMDS plot in August and September (**Figure 5**).

Three subcommunities, composed of abundant, common, and rare ASVs respectively, exhibited similar NMDS patterns. However, the Adonis test revealed that elevational and seasonal changes explained a greater proportion of the variation in the abundant subcommunity, while explaining the least variation in the rare subcommunity (**Supplementary Figure S4**).

### 3.6 Key factors driving elevational and seasonal variations of diazotrophic community

Soil moisture had the greatest impact on diazotrophic communities, followed by soil pH, ammonium, temperature, plant richness, aboveground biomass, and soil C:N ratio (**Table 1**). These factors were well-fitted on the NMDS plots, showing stronger associations with elevational variations than seasonal dynamics (**Figure 4A**).

**Table 1:** Effects of environmental factors on elevational and seasonal variations of diazotrophic community based on partial Mantel test.

	Ammonium	Nitrate	C:N ratio	Soil pH	Moisture	Temperature	Plant richness	Aboveground biomass	Legume biomass
all samples	0.171^***^	0.036	0.079^*^	0.183^***^	0.225^***^	0.115^**^	0.167^***^	0.106^**^	0.048
(a) grouped by month									
Jun.	0.209^**^	0.162	0.293^***^	0.288^**^	0.195^**^	0.527^***^	0.048	0.056	0.085
Jul.	0.036	0.114	0.118	0.034	0.465^***^	0.628^***^	0.120	0.112	0.250^**^
Aug.	0.167^*^	0.073	0.107	0.044	0.220^**^	0.340^**^	0.160^*^	0.113	0.031
Sept.	0.003	0.150	0.022	0.115	0.166^*^	0.577^***^	0.074	0.074	0.131
(b) grouped by elevation									
3200 m	0.110	0.065	0.023	0.059	0.100	0.184^*^	0.154	0.412^***^	0.222^*^
3400 m	0.054	0.056	0.038	0.021	0.221^*^	0.156^*^	0.014	0.235^**^	0.074
3600 m	0.187	0.139	0.049	0.189	0.032	0.329^***^	0.090	0.032	0.117
3800 m	0.058	0.097	0.147	0.008	0.206^*^	0.072	0.124	0.019	0.014
4000 m	0.040	0.127	0.182^*^	0.229^*^	0.113	0.333^**^	0.029	0.109	0.103

When one environmental factor was analyzed, the remaining environmental variables were controlled. Significant values are in bold: ^*^ *P*<0.05, ^**^ *P*<0.01, ^***^ *P*<0.001.

To disentangle the effects of elevation and season, we separately assessed environmental factors influencing elevational and seasonal variations of diazotrophic communities (**Figures 5**, **6**, **Table 1**). Partial Mantel test showed that temperature and moisture consistently influenced elevational variations across the growing season, with temperature having a greater effect. In contrast, key factors driving seasonal dynamics varied at different elevations. At low elevations (3200-3400 m), aboveground biomass was the primary factor, with legume biomass also being significant at 3200 m. However, at 3600 m and 4000 m, temperature was the primary factor. In fact, temperature was a significant factor at almost all elevations. In addition, moisture was the primary factor at 3800 m and also significant at 3400 m. Notably, soil C:N ratio was also significant at 4000 m and increased throughout the growing season at high elevations (**Supplementary Figure S1F**).

In summary, elevational variations were primarily driven by temperature and moisture, whereas seasonal dynamics depended more on plant growth at low elevations and climatic factors at higher sites.

## 4 Discussion

### 4.1 Elevational changes in diazotrophic communities driven by temperature and moisture

Our results indicated that elevational variation significantly affected soil diazotrophic community structures in alpine grasslands throughout the growing season, primarily driven by temperature and soil moisture. Low temperature is one of the limiting factors for BNF in alpine grasslands (Holzmann and Haselwandter, 1988). The higher temperature at low elevation not only enhances nitrogenase activity but also promotes plant growth, thereby altering the composition of soil diazotrophic communities (Wang et al., 2022). For example, low elevations are more favorable for the growth of leguminous plants, which increase the proportion and N fixation activity of symbiotic rhizobia, further raising soil ammonium concentration (Rui et al., 2022; Tang et al., 2024). BNF is an energy-intensive process (Norman and Friesen, 2017), and free-living diazotrophs require additional energy to overcome the toxic effects of oxygen on nitrogenase (Smercina et al., 2019). When available N is sufficient in the environment, free-living diazotrophs tend to avoid N fixation (Reed et al., 2011). Therefore, in soils at 3200 m, where legume biomass and ammonium concentration were highest, the relative abundance of free-living diazotrophs, soil nitrogenase activity (representing non-symbiotic N fixation activity), and *nifH* gene copies were all relatively low there (**Figure 1**).

Soil moisture is another important factor influencing the elevational variation. In mountainous environments, moisture typically follows an inverted V-shaped pattern along elevations (Rahbek, 1995). In our study sites, soil moisture also showed an inverted V-shaped pattern across elevations during the growing season (**Supplementary Figure S1**), as a result of orographic precipitation patterns (Xu et al., 2010). This pattern resulted in a non-linear variation of diazotrophic communities along the elevational gradient, with the most significant differences observed between the communities at 3600 m and those at 3200 m (**Figure 4**). The higher moisture at 3600 m hindered the growth of leguminous plants, leading to a very low relative abundance of rhizobia. Moreover, the relatively anaerobic and moist environment favors anaerobic free-living diazotrophs by reducing oxygen toxicity to nitrogenase (Smercina et al., 2019). This led to the highest relative abundance of *Geobacter*, soil nitrogenase activity, and *nifH* gene copies.

The effects of temperature and moisture on the elevational differentiation of diazotrophic communities are consistent with our previous study sampled in Sept. 2020 (Rui et al., 2022), indicating that diazotrophic communities generally exhibit similar elevational distribution patterns throughout the growing season. This study extends these findings by demonstrating that this pattern holds true across the entire growing season, highlighting the consistent role of temperature and moisture in regulating not only the species composition of diazotrophic communities but also the significant structural differences between communities at different elevations.

### 4.2 Seasonal dynamics of diazotrophic community driven by plant dynamics and microclimatic factors

Seasonal variations in diazotrophic communities of alpine grasslands across different elevations exhibited significant differences, primarily driven by seasonal dynamics of temperature, soil moisture, and plant dynamics. At lower elevations (3200 m and 3400 m), the dominant factor influencing seasonal dynamics of diazotrophic communities was the aboveground biomass. Additionally, legume biomass was also one of the significant influencing factors at 3200 m. This may be attributed to the greater seasonal variation in plant and legume biomass at lower elevations, particularly the significant difference between June and the other months of the growing season. Plant growth significantly influences soil microbial communities in grasslands through several mechanisms, such as changes in root exudates, nutrient uptake patterns, and litter input (Che et al., 2020; Steinauer et al., 2023). In our study, June marked the early growing season, during which plant biomass, including that of legumes, was lower at all elevations but significantly increased from July to September at lower elevations. At 3600 m and 4000 m, temperature was the most important factor, significantly influencing the seasonal changes in diazotrophic communities at all elevations except 3800 m. Seasonal variations in soil moisture were relatively minor, significantly affecting only the 3400 m and 3800 m sites. Therefore, seasonal changes in plant biomass and soil micoclimate factors, especially temperature, drive the dynamics of diazotrophic communities across elevations.

The soil nitrogenase activity and *nifH* gene copies were the highest in June (**Figure 1**), which indicated that free-living N fixation may play important roles in the early grow season. In habitats devoid of leguminous plants, free-living N fixation may represent the predominant form of BNF (Reed et al., 2011). Our sequencing results suggest that the alpine grasslands were primarily dominated by free-living diazotrophs in June, due to slower plant growth and the lack of legumes. From July to September, the contribution of associative N fixation increases. However, the high proportion of symbiotic diazotrophs in soils does not necessarily indicate high symbiotic N fixation in June, as this process occurs within root nodules. Studies have shown that rhizobia are released into the soil from ruptured root nodules at the end of the growing season (Poole et al., 2018; Krutylo and Nadkernychna, 2023). They live saprophytically in soils during the non-growing season, and infect legume roots again in the next growing season. This may explain the higher proportion of rhizobia in the soil in June.

The C:N ratio is an important indicator regulating N fixation activity of non-symbiotic diazotrophs, with a high C:N ratio favoring N fixation (Reed et al., 2011; Inomura et al., 2018). We noted considerable variations in the soil C:N ratio across elevations, with that at 3200 m being significantly lower than at higher elevations (**Supplementary Figure S1F**). This suggests a relatively plentiful supply of N sources at lower elevations, perhaps attributable to increased symbiotic N fixation activity. As elevation ascends, the C:N ratio also rises while plant biomass diminishes, signifying a reduction in N availability. This highlights the important role of free-living diazotrophs in BNF at elevated elevations, especially under colder temperature circumstances. The temporal patterns of soil C:N ratio during the growing season differed between low and high elevations. At the low elevation (3600 m), the soil C:N ratio rose from June to August but declined to a lower level after September. This suggests that symbiotic and associative N fixation could meet plant N requirements throughout the growing season, thereby maintaining N balance. In contrast, the soil C:N ratio at high elevations (3600–4000 m) gradually increased from June to September, indicating that the rate of free-living N fixation could not keep up with plant N demand, exacerbating N limitation. The observed differences likely result from two key factors. First, the warmer conditions at lower altitudes enhance nitrogenase activity. Second, the inherent efficiency disparity between free-living and symbiotic N fixation. While symbiotic N fixation (e.g., rhizobia-legume associations in root nodules) achieves highly efficient N fixation, free-living diazotrophs must expend significantly more energy to mitigate oxygen toxicity, leading to substantially higher metabolic costs for N fixation (Smercina et al., 2019).

### 4.3 Contributions of abundant and rare species to diazotrophic community diversity

The diversity of diazotrophic communities depends not only on the richness and species composition of the community but also on the dynamics of abundant and rare species. We found that the beta diversity of diazotrophic communities was more strongly influenced by abundant species. Subcommunities composed of just 192 abundant ASVs effectively represent the beta diversity of the entire diazotrophic communities (**Supplementary Figure S4**). Abundant genera, such as *Geobacter*, *Mesorhizobium*, and *Azospirillum*, shaped the beta diversity of diazotrophic communities under different elevational and seasonal conditions, with their relative abundance changes driving notable differences in community composition along elevations. For example, *Geobacter* was predominant at middle elevations, while *Mesorhizobium* dominated at lower elevations, with both groups showing a stronger presence in June. Interestingly, compared to rare species, the subcommunity composed of abundant species can be better explained by changes in elevation and season (**Supplementary Figure S4**), suggesting that the species composition of abundant species may be more sensitive to environmental changes.

Rare species had a major impact on the alpha diversity of diazotrophic community, with their relative abundance being notably higher at middle elevations compared to other elevations. These rare species primarily belonged to *Geobacter* and unclassified Alphaproteobacteria. Interestingly, the number of ASVs belonging to *Geobacter* also followed an inverted V-shaped pattern along the elevational gradient, positively correlated with its relative abundance. Geobacter possesses remarkable adaptive and evolutionary capabilities (Summers et al., 2012), which may explain the exceptionally high ASV richness of this genus at mid/high elevations. The relatively anaerobic soils at mid-elevations are less conducive to the majority of plants, including legumes, leading to the lowest relative abundances of both symbiotic and associative diazotrophs. However, the richness of rare diazotrophic species was highest in this zone. These rare species might play a potential role in the stability and adaptability of the community, possibly acting as key players in community recovery and ecological adaptation (Ma et al., 2024). The presence of rare species may enhance the functional redundancy of diazotrophic communities, particularly at higher elevations, where they may contribute N fixation through distinct physiological functions in N-limited environments. For example, *Geobacter* is considered a successful N fixer in nutrient-deficient conditions, utilizing ferric iron (Fe^3+^) as the electron acceptor (Bazylinski et al., 2000). Therefore, rare species may play a crucial role in maintaining community diversity and significantly contribute to the ecological adaptation of diazotrophic community (Jain et al., 2014). The interaction between abundant and rare species is complex and multi-layered, reflecting not only the impact of environmental gradients on diazotrophic community structure but also the interaction and functional complementarity between different species communities in ecosystems.

## 5 Conclusion

This study highlights the dynamic characteristics of diazotrophic communities in alpine grasslands, accentuating their elevational and seasonal variations driven by soil microclimate factors and plant dynamics. Our findings indicated that diazotrophic communities typically display uniform elevational distribution patterns throughout the growing season, predominantly affected by temperature and moisture. Seasonal variations were more pronounced at lower elevations, driven by vegetation, while microclimate factors, particularly temperature, held greater significance at higher elevations. Additionally, *nifH* gene abundance and soil nitrogenase activity were higher in the early growing season, suggesting that free-living N fixation may play a crucial role in BNF before legumes and grasses reach maturity in grasslands. We also find that abundant species were key contributors to diazotrophic beta diversity, whereas rare species were instrumental in determining alpha diversity. These findings underscore the complexity of diazotrophic community dynamics and emphasize the need for a deeper understanding of the roles of both abundant and rare species in ecosystem functioning. However, a limitation of this study is that only soil nitrogenase activity was monitored, which primarily reflects the nitrogenase activity of free-living N fixation in soils. Future research should also monitor plant-associated N fixation rate in alpine grassland ecosystems.

## Data Availability

The datasets presented in this study can be found in online repositories. The names of the repository/repositories and accession number(s) can be found below: https://ngdc.cncb.ac.cn, CRA021903.
