# Supplementary material for: Soil microclimate and vegetation dynamics shape elevational and seasonal variations of diazotrophic communities in alpine grasslands
Source: Front Plant Sci. 2025 Sep 15;16:1587343. doi: 10.3389/fpls.2025.1587343 (PMC12477229; doi:10.3389/fpls.2025.1587343)
Supplement: Supplementary file 1 [file DataSheet1.docx]

Supplementary Material

Soil Microclimate and Vegetation Dynamics Shape Elevational and Seasonal Variations of Diazotrophic Communities in Alpine Grasslands

Junpeng Rui, Xiaojian Long, Xuemiao Wang, Xinyu Xiong, Jianxiao Zhu

*** Correspondence:** Junpeng Rui: [ruijp@lzu.edu.cn](mailto:ruijp@lzu.edu.cn)

**Supplementary Figures**


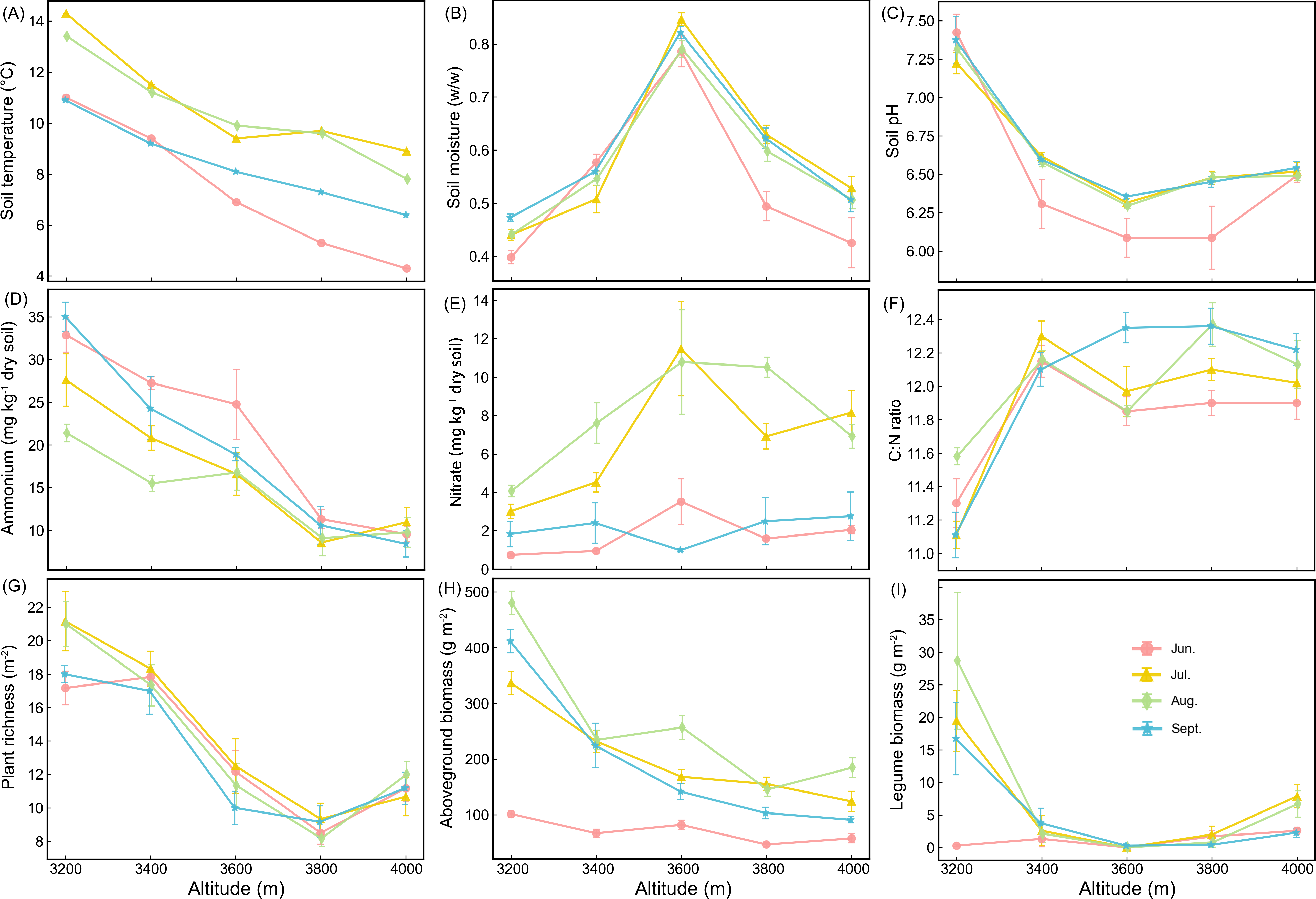


Figure S1. Elevational changes of environmental factors during the growing season. Error bars represent standard deviations (n = 6). Some data are taken from (Rui et al., 2023), previously published as supplementary materials.


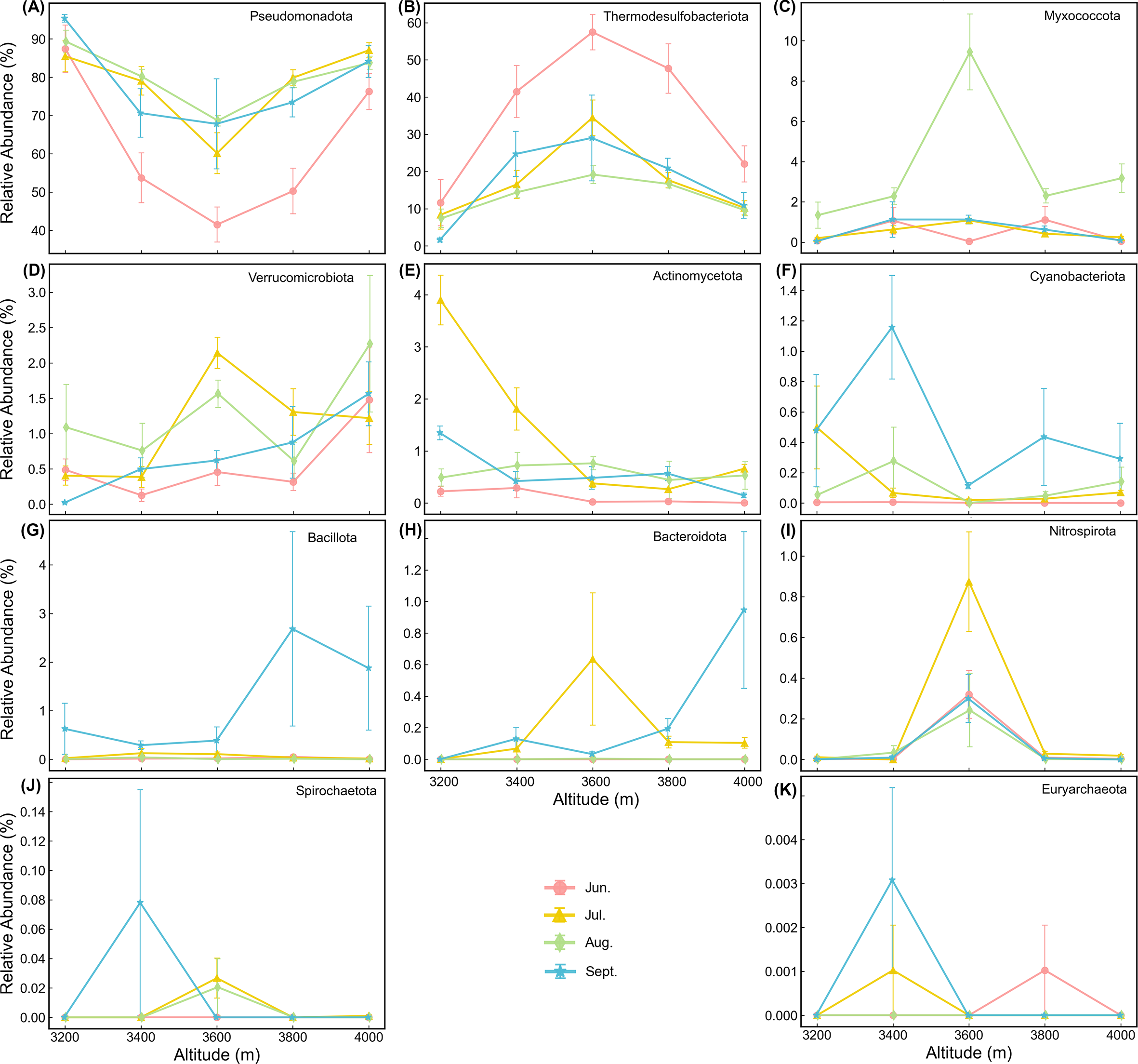


Figure S2. Elevational changes of diazotrophs during the growing season at the phylum level. Error bars represent standard errors (n = 6).


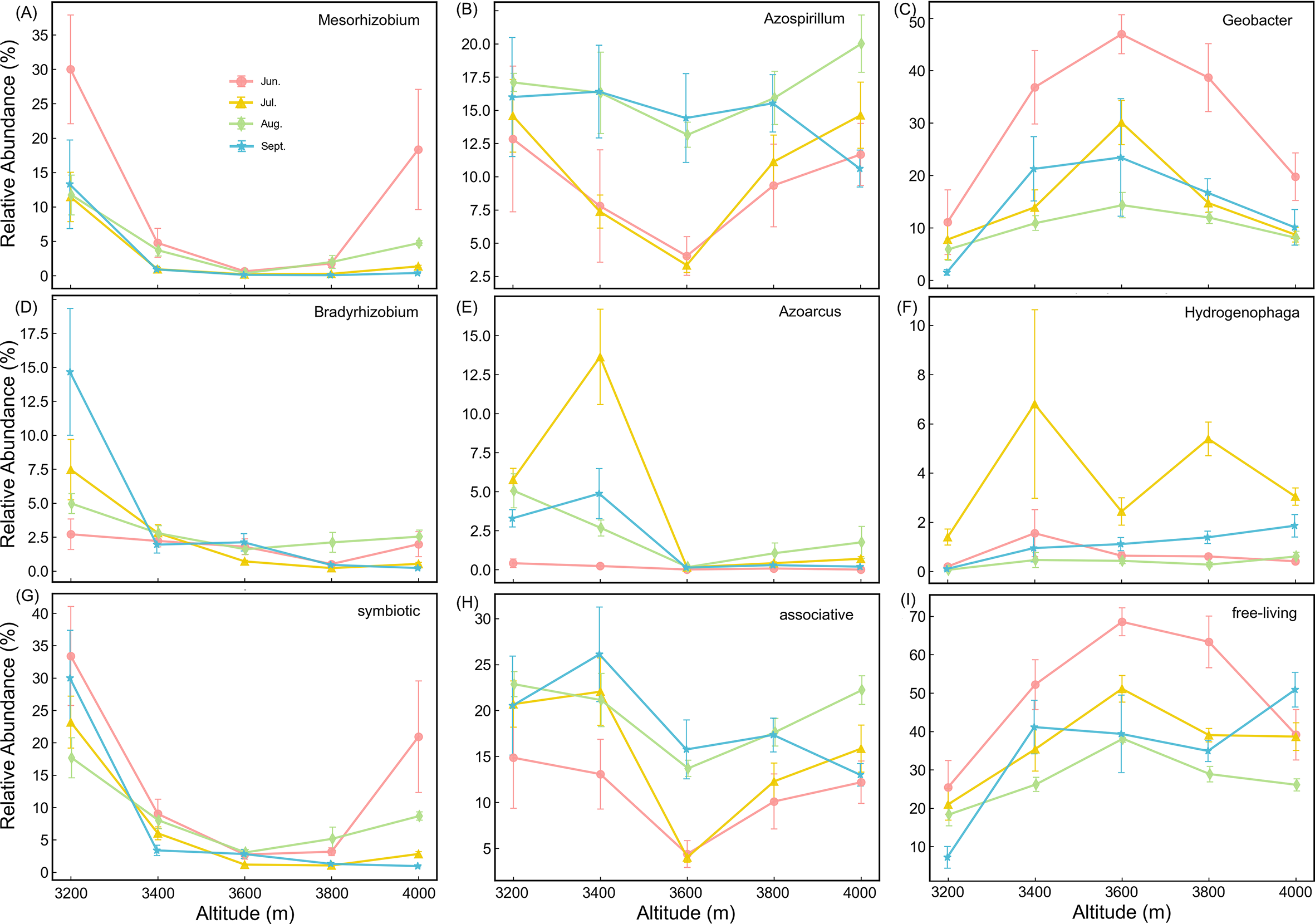


Figure S3. Elevational changes of different types of diazotrophs during the growing season. (A-F) The top 6 genera. (G-I) Total relative abundances of ASVs affiliated to symbiotic, associative, and free-living diazotrophs. Error bars represent standard errors (n = 6).


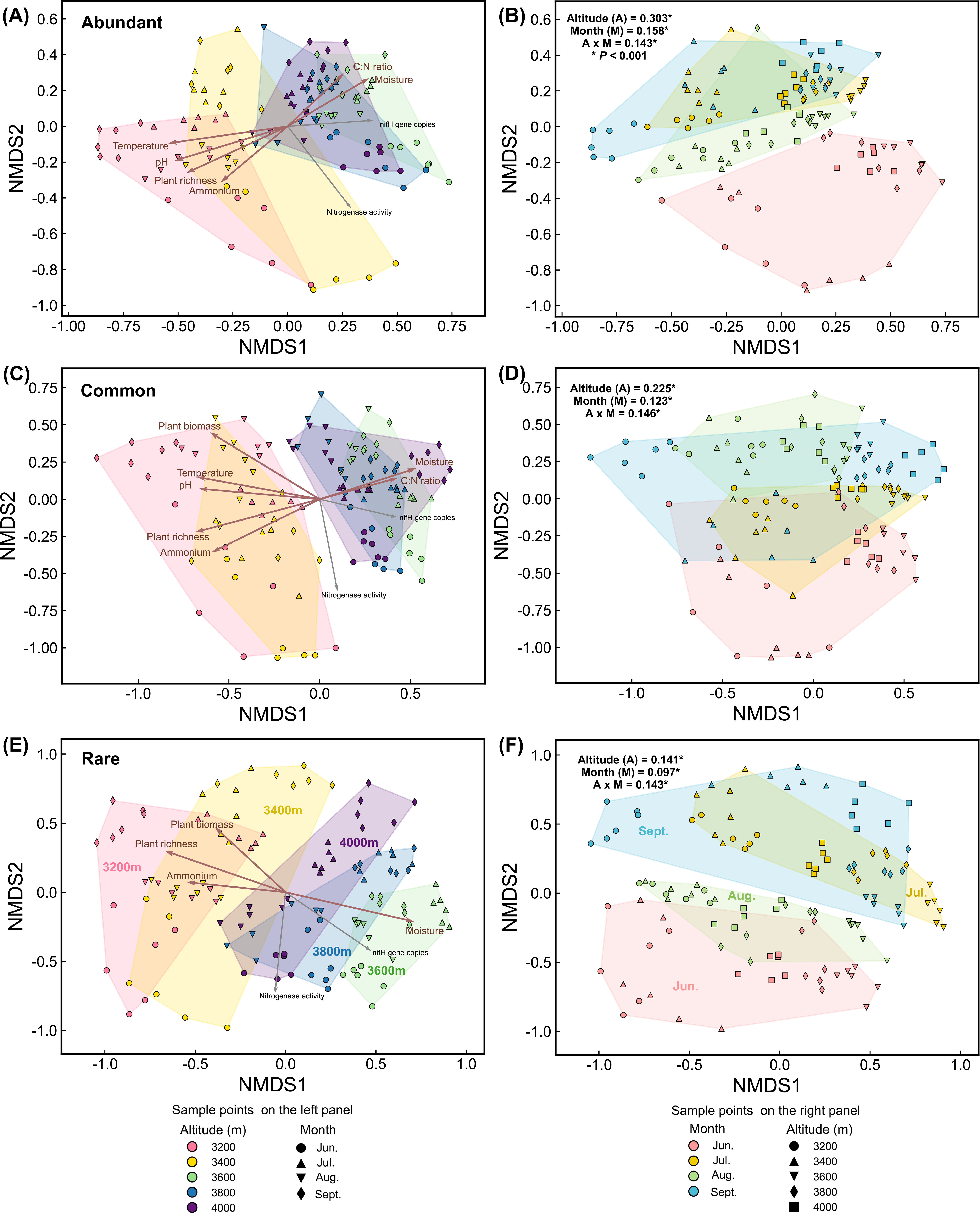


Figure S4. NMDS patterns and Adonis test results for subcommunities consisted of (A-B) abundant ASVs, (C-D) common ASVs, and (E-F) rare ASVs. Sample points in the left panel are colored by elevation, while those in the right panel are colored by month.

Table S1. All diazotrophic genera and their N fixation types.

| Genus | Mean relative abundance (%) | N fixation type |  | Genus | Mean relative abundance (%) | N fixation type |
| --- | --- | --- | --- | --- | --- | --- |
| *Azospirillum* | 12.60619 | associative |  | *Leptothrix* | 0.23063 | unknown |
| *Mesorhizobium* | 5.34634 | symbiotic |  | *Sphaerotilus* | 0.1914 | unknown |
| *Bradyrhizobium* | 2.71768 | symbiotic |  | *Aromatoleum* | 0.09928 | free-living |
| *Salipiger* | 0.37375 | free-living |  | *Propionivibrio* | 0.05634 | free-living |
| *Magnetospirillum* | 0.18205 | free-living |  | *Azospira* | 0.03456 | free-living |
| *Rhodovulum* | 0.16228 | free-living |  | *Aquabacterium* | 0.02768 | unknown |
| *Skermanella* | 0.16222 | unknown |  | *Rubrivivax* | 0.00519 | free-living |
| *Rhizobium* | 0.14093 | symbiotic |  | *Candidatus Accumulibacter* | 0.00406 | unknown |
| *Methylobacterium* | 0.07175 | free-living |  |  |  |  |
| *Methylocystis* | 0.06671 | free-living |  | *Halorhodospira* | 0.00272 | free-living |
| *Novosphingobium* | 0.05264 | free-living |  | *Pseudomonas* | 0.00118 | associative |
| *Gluconacetobacter* | 0.03066 | associative |  | *Marinobacterium* | 0.00031 | free-living |
| *Beijerinckia* | 0.00339 | free-living |  | *Klebsiella* | 0.0001 | associative |
| *Xanthobacter* | 0.00051 | free-living |  | *Geobacter* | 17.6421 | free-living |
| *Nitrospirillum* | 0.00026 | associative |  | *Desulfuromonas* | 0.2481 | free-living |
| *Rhodopseudomonas* | 0.0001 | free-living |  | *Desulfovibrio* | 0.02814 | free-living |
| *Methyloceanibacter* | <0.0001 | free-living |  | *Desulfobulbus* | 0.00072 | free-living |
| *Azoarcus* | 2.04428 | associative |  | *Anaeromyxobacter* | 1.26375 | free-living |
| *Hydrogenophaga* | 1.4893 | free-living |  | *Frankia* | 0.63161 | symbiotic |
| *Dechloromonas* | 1.12949 | free-living |  | *Paenibacillus* | 0.10712 | associative |
| *Azonexus* | 1.10548 | free-living |  | *Nostoc* | 0.07343 | free-living |
| *Methyloversatilis* | 0.93938 | free-living |  | *Scytonema* | 0.01043 | free-living |
| *Polaromonas* | 0.70909 | free-living |  | *Trichodesmium* | 0.0093 | free-living |
| *Rhodoferax* | 0.36579 | free-living |  | *Tolypothrix* | 0.00267 | free-living |
| *Herbaspirillum* | 0.32369 | associative |  | *Cylindrospermum* | 0.00077 | free-living |
| *Paraburkholderia* | 0.30392 | symbiotic |  | *Cyanothece* | 0.00026 | free-living |
| *Sideroxydans* | 0.29482 | unknown |  | *Paludibacter* | 0.00144 | unknown |
| *Burkholderia* | 0.28093 | associative |  | *Arcticibacter* | <0.0001 | unknown |

**Reference for supplementary materials:**

Rui, J., Zhao, Y., Cong, N., Wang, F., Li, C., Liu, X., Hu, J., Ling, N., and Jing, X., 2023. Elevational distribution and seasonal dynamics of alpine soil prokaryotic communities. Front Microbiol 14, 1280011.
